# Supplementary material for: The RNA cargo of plasma-derived extracellular vesicles in mCRPC patients captures cancer cells and tumor microenvironment signals
Source: bioRxiv. 2026 Sep 7:2026.09.03.747025. Preprint. [Version 1] doi: 10.64898/2026.09.03.747025 (PMC13622529; doi:10.64898/2026.09.03.747025)
Supplement: Supplementary file 1 [file NIHPP2026.09.03.747025V1-supplement-1.pdf]

bioRxiv preprint doi: https://doi.org/10.64898/2026.09.03.747025; this version posted September 7, 2026. The copyright holder for this preprint (which was not certified by peer review) is the author/funder, who has granted bioRxiv a license to display the preprint in perpetuity. It is made available under a CC-BY 4.0 International license.

Supplementary methods

1 Transcripts Integrity Number (TIN)

To assess the integrity of plasma-derived transcripts, the TIN was used as a metric, which evaluates the uniformity of coverage along the length of each transcript[22]. TIN values were computed at the transcript level and subsequently collapsed to the gene level by selecting, for each gene, the transcript with the highest TIN value, thereby retaining the best-supported estimate of transcript integrity.

bioRxiv preprint doi: https://doi.org/10.64898/2026.09.03.747025; this version posted September 7, 2026. The copyright holder for this preprint (which was not certified by peer review) is the author/funder, who has granted bioRxiv a license to display the preprint in perpetuity. It is made available under a CC-BY 4.0 International license.

2 Proxy of Fragment Length (PFL)

An additional metric, termed Proxy of Fragment Length (PFL), was used as an orthogonal measure to TIN for assessing transcript integrity in the SE150 sequencing protocol. PFL is based on the read mapping length (RML) from transcriptomic BAM files, with soft-clipped bases excluded when evaluating read length. Specifically, from each file, by using a custom bash script, the CIGAR was extracted and the RML was calculated by using the GenomicAlignments v1.36.0 R package, excluding soft-clipped bases with the *cigarWidthAlongQuerySpace* function. To collapse transcript-level information to the gene-level, PFL is computed as the ratio of the per-gene median RML to the minimum of either the median transcript length from the hg38 annotation or 150 bp (the maximum read length used for the single-end sequencing protocol):

$$PFL = \left(\frac{median\ RML}{\min(median\ transcript\ length, 150)}\right) * 100$$

3 Nanopore computational workflow for read length analysis

Long-read EV-RNAseq fastq data was downloaded using the fasterq-dump script from SRA toolkit v2.10 using an SRR accession list downloaded from GEO NCBI (https://www.ncbi.nlm.nih.gov/geo/) with accession number GSE225471. Reads adapters were trimmed by using Porechop v0.2.3, then reads were filtered for quality and minimum length by using Chopper v0.8[76] with the following parameters: -q 7 -l 15. The resulting reads were mapped into the human genome hg38 by using minimap2 v2.28[77] with parameters: -ax splice. Using the resulting BAM files, the mean and standard deviation of the read lengths were calculated for each sample and used as input for pseudomapping performed with Kallisto v0.44[33], to assign reads to transcripts, with the following parameters: --single --single-overhang --fr-stranded. To compare the resulting read lengths between cells and EVs, a Wilcoxon's Mann-Withney two sided test was used.

4 scRNA-seq reference contruction for BayesPrism deconvolution

4.1 scRNA-seq data integration

PCa scRNA-seq data[25] (N = 12) and PBMC scRNA-seq data[26] (N = 12) were integrated using Seurat v4.3, following published guidelines[78]:

bioRxiv preprint doi: https://doi.org/10.64898/2026.09.03.747025; this version posted September 7, 2026. The copyright holder for this preprint (which was not certified by peer review) is the author/funder, who has granted bioRxiv a license to display the preprint in perpetuity. It is made available under a CC-BY 4.0 International license.

Data preparation: The PBMC dataset was streamlined using *DietSeurat*, retaining only count and data slots. The PCa dataset was filtered to include tumor samples only.

Feature selection for integration: *SelectIntegrationFeatures* was used to identify the top 10,000 most variable genes across both datasets, optimizing feature overlap and robustness.

Preprocessing: Each dataset was normalized using *NormalizeData* with the Relative Counts (RC) method (scaling factor = 1,000,000). *FindVariableFeatures* (method = "vst") was applied separately to each dataset to select the top 10,000 variable features. Datasets were then scaled and processed using PCA (*RunPCA*). Raw counts were retained for downstream BayesPrism deconvolution.

Integration: *FindIntegrationAnchors* and *IntegrateData* were used to integrate the datasets using the shared 10,000 most variable gene set, applying log-normalization.

Cell type harmonization: Cell type labels were standardized across datasets (e.g., merging “NK cells” and “nk cell”) to ensure consistency. Harmonized Cell types have been used as Cell Type, while the tissue of origin has been used as cell state.

4.2 Gene filtering based on plasma EV-RNA data

The genes have been filtered to be represented in the PRIME Enza cohort and HDs (see **Materials and Methods** section **4.1**) with 5 cpm in at least 10% of the 135 samples. The 15,432 genes selected were intersected with the 10,000 most variable genes from scRNA-seq integrated data, obtaining 6,064 genes for subsequent deconvolution which has been applied to PRIME Enza cohort.

4.3 Testing for batch effect in scRNA-seq reference

Optimal clustering parameters were determined by sampling random subsets of n=5,000 cells from the constructed scRNA-seq reference and applying the shared nearest neighbor (SNN) algorithm at various resolution values. Clustering outcomes were assessed across these resolution values with Silhouette scores, calculated for each cluster to evaluate cohesion. The maximum median Silhouette score allowed to choose the best resolution for clustering: 0.1. The clustering with the optimal resolution was visualized using UMAP, with cells colored by cluster assignment (**Figure S3.1A-B**). Clustering quality was evaluated by calculating the Adjusted Rand Index (ARI) with

bioRxiv preprint doi: https://doi.org/10.64898/2026.09.03.747025; this version posted September 7, 2026. The copyright holder for this preprint (which was not certified by peer review) is the author/funder, who has granted bioRxiv a license to display the preprint in perpetuity. It is made available under a CC-BY 4.0 International license.

respect to assay and cell type. A higher ARI with cell type (0.44) than assay (0.15) suggested minimal batch effects related to assay differences, indicating robust clustering by cell type.
